# Animal Models of Type III Secretion System-Mediated Pathogenesis

**DOI:** 10.3390/pathogens8040257

**Published:** 2019-11-22

**Authors:** Julia A. Hotinger, Aaron E. May

**Affiliations:** Department of Medicinal Chemistry, Virginia Commonwealth University, Richmond, VA 23284, USA; hotingerja@vcu.edu

**Keywords:** type III secretion system, pathogenesis, virulence, animal models

## Abstract

The type III secretion system (T3SS) is a conserved virulence factor used by many Gram-negative pathogenic bacteria and has become an important target for anti-virulence drugs. Most T3SS inhibitors to date have been discovered using in vitro screening assays. Pharmacokinetics and other important characteristics of pharmaceuticals cannot be determined with in vitro assays alone. In vivo assays are required to study pathogens in their natural environment and are an important step in the development of new drugs and vaccines. Animal models are also required to understand whether T3SS inhibition will enable the host to clear the infection. This review covers selected animal models (mouse, rat, guinea pig, rabbit, cat, dog, pig, cattle, primates, chicken, zebrafish, nematode, wax moth, flea, fly, and amoeba), where T3SS activity and infectivity have been studied in relation to specific pathogens (*Escherichia coli*, *Salmonella* spp., *Pseudomonas* spp., *Shigella* spp., *Bordetella* spp., *Vibrio* spp., *Chlamydia* spp., and *Yersinia* spp.). These assays may be appropriate for those researching T3SS inhibition.

## 1. Introduction

Antibiotics have revolutionized how the medical community treats bacterial infections and have contributed to the overall increase in life expectancy throughout the world [1]. A large and growing problem, however, is that antibiotic resistance threatens to make our supply of antibiotics ineffective [2]. An emerging strategy for combating pathogenic bacteria is to suppress their ability to cause and sustain an infection. These anti-virulence drugs would target pathogenesis pathways and virulence factors in pathogenic bacteria in order to stop or slow infection [3]. The type III secretion system (T3SS) is a virulence factor commonly used by pathogenic Gram-negative bacteria [4,5,6]. Some examples of bacteria which produce T3SSs include, but are not limited to, enteropathogenic and enterohemorrhagic *Escherichia coli* (EPEC and EHEC, respectively) [7,8,9], *Salmonella* spp. [6], *Pseudomonas* spp. [5], *Shigella* spp. [10], *Bordetella* spp. [11], *Vibrio* spp. [12], *Chlamydia* spp. [13], and *Yersinia* spp. [14,15] The T3SS is a needle-like apparatus that allows for the secretion of virulence proteins, called effectors, into the host cell [16,17]. The needle spans the bacterial membranes and the host cell membrane, allowing for direct secretion from the pathogen into the host. These secreted effector proteins reprogram the host’s machinery to allow for colonization. In addition to reprograming host machinery (*Salmonella* spp., *Shigella* spp.) [18,19], some effectors kill the target cells (*Yersinia* spp., *Pseudomonas* spp.) [20,21].

The T3SS is an attractive anti-virulence target, because many T3SS knockout strains have attenuated virulence [12,22,23,24,25,26,27,28,29,30,31]. Efforts have gone into screening for T3SS inhibitors, and a common theme among them is a lack of toxicity to the pathogen [22,32,33]. This is an important feature of these inhibitors, because it means they do not exert selective pressure. This should reduce the rate of formation of resistance to these agents [34].

## 2. Animal Models

Many animal models have been used to study Gram-negative pathogens that use a T3SS (Table 1) [35,36,37,38,39,40,41,42,43,44,45,46,47,48,49,50,51,52,53,54,55,56,57,58,59,60,61,62,63,64,65,66,67,68,69,70,71,72,73,74,75,76,77,78,79,80,81,82,83,84,85,86,87,88,89,90,91,92,93,94,95,96,97,98,99,100,101,102,103,104,105,106,107,108,109,110,111,112,113,114,115,116,117,118]. Assays have been developed that study overall survival, bacterial loads, virulence, and histopathological characteristics of pathogenesis. These assays can vary significantly between animal models. For example, some bacterial load assays that require whole animal homogenization cannot be performed on larger animals. On the other hand, organ homogenization or xenotransplants cannot be performed in small animals such as insects. This section covers selected in vivo techniques to study the T3SS.

Mice are the most common rodent used to study T3SS pathogenesis [35,36,37,38,39,40,41,42,43,44,45,46,47,48,49,50]. They are small, inexpensive, and well described in comparison to other models. The ease of genetic engineering is another advantage of using mice. Transgenic mice can be tailored to have increased susceptibility to specific bacterial infections [119]. This allows the murine model to cover the widest variety of disease states.

The use of animals such as rabbits, cats, and dogs comes with a more stringent set of ethical ramifications due, in part, to their use as companion animals by the public [120]. Testing with these animals can garner attention from the press and those against animal testing in general. Many specific species in this category are considered United States Department of Agriculture (USDA) protected and require additional approvals and protocol requirements when using them [121]. These models, however, are very important for testing specific pathogens. For example, rabbits and guinea pigs provide the most cost-effective method to study shigellosis, which otherwise is most commonly studied in primates.

Non-human primates are the best and most accurate animal model of human infection [85,86,87,88,89,90,91,92,93] and are often used in preclinical trials [122]. Similar to companion animals, non-human primates are also USDA protected, although the approvals and protocol requirements differ. Unfortunately, they are very expensive to work with and are routinely used in multiple studies. A high variability between animals is possible due to monkeys having different experimental histories. For example, in a study of Shigella infection in rhesus macaques, nine of fifteen monkeys were historically exposed to *plasmodium*, *leishmania*, dengue virus [91]. A user of primate models must be aware of how history can influence susceptibility to infection.

Industrial animals such as pigs, chickens, and cattle are of importance because they are often carriers of pathogens such as *Salmonella* and *E. coli* [75,76,81,82,83,94,95]. The food industry often uses antibiotics to prevent infection and for growth promotion. This causes antibiotic resistance genes to form at high rates among the bacterial populations within these animals and their surrounding environment [123,124]. Research performed using these models can expand understanding of foodborne pathogens and help prevent outbreaks.

Fish models can allow for a higher degree of control than those using open cages [29,98,99,100,101,125]. Aquariums allow for the control of temperature, oxygen content, pathogen load, and any other reagent. They are also cost effective to work with and most fish can also be housed individually. Fish, like the zebrafish, are natural hosts of *Vibrio* spp. and are used to study this pathogen [125]. Fish are not a good model for many other pathogens due to their lack of similarity in clinical symptoms with humans. They are convenient for early studies of potential pharmaceuticals, but mammals should be used later.

Invertebrates such as worms, insects, and amoebas can be used as animal models [102,103,104,105,106,107,108,109,110,111,112,113,114,115,116,117,118]. They provide the ability to readily use large numbers of subjects due to fast reproduction, ease of handling, and small size. Invertebrate groups may contain large (>50) numbers of animals per group, whereas mammalian groups typically range from three to ten [114]. Invertebrates can even be used in semi-high throughput screening [126].

### 2.1. Survival Assays

Survival assays are widely used in the field of infectious disease. The animal is injected or inoculated with a pathogen to cause infection. The length of time for the onset of symptoms or death is then measured. This process can then be repeated with an agent to treat the infection to judge its efficacy. The inhibitors are given either at the time of challenge, or at a later timepoint. This allows for observation of symptom onset, symptom prevalence, or changes in lifespan when inhibitors are used. The Abe laboratory treated mice with *Citrobacter rodentium*, a common EPEC pathogen model, either with or without the natural product T3SS inhibitor aurodox [36]. They first showed with in vitro assays that aurodox strongly inhibited hemolysis of red blood cells by EPEC and inhibited secretion of the effectors EspB, EspF, and Map. It was then used in vivo and it was discovered that aurodox reduced the severity of infection caused by *C. rodentium* and allowed all the mice to survive to the experimental endpoint. This was an important result because it was the first time that a T3SS inhibitor had been shown to be effective as an anti-infective agent [36].

More recently, the Deng and Luo groups tested the T3SS inhibitor thymol in a survival assay using the pathogen *Salmonella enterica* serovar Typhimurium (*S*. Typhimurium) [41]. They found that thymol treatment allowed the mice to survive an otherwise lethal dose of pathogen, and alleviated symptoms of infection such as weight loss. The Deng laboratory has also studied the effects of syringaldehyde, another natural product T3SS inhibitor, on *S*. Typhimurium [39]. They treated mice with 100 mg/kg syringaldehyde three times on the day before infection, as well as for five days after infection. Mice treated with syringaldehyde had a 60% survival rate. The untreated mice all died by day 7 of the experiment, which shows that syringaldehyde had a significant impact on survival. They were also able to observe that syringaldehyde specifically inhibited the expression of the T3SS effector proteins SipA, SipB, and SipC.

Survival studies can also be performed by comparing wild-type to mutant pathogen strains. This can allow for the importance of specific genes to be judged. For example, the Swietnicki laboratory created a YscN knockout strain of *Yersinia pestis*, the causative agent of the bubonic plague [50]. This gene encodes an ATPase that energizes the T3SS and allows for the delivery of effector proteins through the needle. A survival assay using subcutaneous delivery of *Y. pestis* to mice was used. A typical LD_50_ for this pathogen had been reported between 1–2 colony-forming units (CFU). The *Y. pestis* YscN knockout was tested up to 32,000,000 CFU without a single mouse dying from the infection, and none of the mice showed signs of discomfort or symptoms of disease. The knowledge that YscN was critical to the infectivity of *Y. pestis* and a potential therapeutic target led the authors to perform a virtual screen for YscN inhibitors. The top 37 compounds from the screen were purchased and tested in vitro to determine their IC_50_ values for ATP hydrolysis by YscN. They also observed inhibition of YopE effector secretion. A HeLa cell invasion assay was performed to determine virulence after treatment with each compound. These studies led to the discovery of three lead compounds that will be used for further investigation.

#### Killing Assays

Survival assays are often called killing assays when done in insects or other small invertebrates such as roundworms [108,127], locusts [128], moths [108], flies [129], and amoebas [117]. There are two major types of killing assays, fast killing and slow killing. The major difference between fast and slow killing assays is the time it takes for animals to die, with slow killing assays requiring multiple days and fast killing assays only hours. This difference in timing is due to the way that the animal is infected with the bacteria of interest and/or the conditions that are maintained after infection. There are, however, multiple ways to define these terms. For example, Park and coworkers state that when *Caenorhabditis elegans* is maintained on low-osmolarity or low-nutrient media, the assay is considered slow killing, while if they are on high-osmolarity media, it is fast killing [130,131]. They can also be defined by the cause of death. Simple infection causes slow killing, while toxins produced by bacteria can result in fast killing [102]. Another definition is used in *Drosophila melanogaster* research; there are feeding assays (slow) and pricking or nicking assays (fast). These are defined by the way that bacteria are introduced to the animal. In pricking assays, a needle that has been dipped in bacteria is used to prick the fly, whereas in feeding assays, the bacteria are provided in the food [132]. In this review, assays will be defined in a similar manner to fly research. Fast killing assays are defined as assays where the bacteria are intentionally inoculated into the animal in order to ensure the same amount of bacteria reaches the site of infection. In slow killing models, the pathogenic bacteria are provided in the environment, either in media or in the animal’s food supply, and allowed to spread naturally.

The Drenkard laboratory used the larvae of the wax moth, *Galleria mellonella*, in a fast killing assay to study the T3SS pathogenesis of *Pseudomonas aeruginosa* by comparing strains with different effector proteins knockouts [108]. They cleaned the larvae of any external bacteria to reduce the possibility of contamination. Each larva was then injected with varying amounts of *P. aeruginosa*. For the next four days, the larvae were incubated in 10-cm plates and the number of dead larvae were counted. LD_50_ values were determined for each knockout strain. The authors used this assay to study the effects of double and triple knockout combinations of the known T3SS effector proteins ExoS, ExoT, ExoU, and ExoY. They found that only ExoT and ExoU play significant roles in *G. mellonella* killing, and only one of the two is needed to obtain levels of killing on par with wild-type *P. aeruginosa*.

Slow killing assays typically use worms or larvae. Harmer and coworkers studied *P. aeruginosa* virulence on the roundworm *Caenorhabditis elegans* [104]. In this assay the worms were externally sterilized before being transferred into a *P. aeruginosa*-seeded plate. The dead worm count was taken daily to determine the LT_50_, or the time at which 50% of the worms were killed. This allowed for the worms to become infected and die off in a more natural fashion than if they had been directly inoculated with the pathogen. The group used human isolates of *P. aeruginosa* and compared virulence factors expressed in each isolate. These virulence factors were then correlated to pathogenesis. They found a significant decrease in virulence in one group of isolates in which a genomic fragment containing *exoY* gene, which encodes T3SS adenylate cyclase effector, as well as other genes, was lost. A main advantage of slow killing assays is that the pathogen’s infectivity can be determined. These killing assays have considerable potential for being used to test T3SS inhibitors because their ability to prevent infection (slow killing) or treat infection (fast killing) can be tested individually.

### 2.2. Bacterial Load Determinations

Measuring bacterial load counts is commonly used in conjunction with survival assays. They are used to determine the number of bacteria in a system, which correlates to the severity of infection in the host. Homogenization methods require the death of the animal in order to obtain a result, subsequently preventing tracking of infection over time. Organ homogenization is done in larger animals, while whole animal homogenization is done in invertebrates. These differences are to balance sample size with accuracy of bacterial load. Sample collection refers to taking biological samples at multiple timepoints in order to track the severity of infection. All three of these methods are easy to perform and have become the most common methods of bacterial load determination.

#### 2.2.1. Organ Homogenization

The most common bacterial load assay is organ homogenization. It is performed after the animal is sacrificed [38,43,46]. The animal’s infected organs are harvested, cleaned, and homogenized in order to be plated using serial dilution. CFUs can be counted after incubation. Organ homogenization allows the fate of pathogenic bacteria to be determined when an inhibitor is given. Since T3SS inhibitors do not directly kill bacteria, these assays can allow the user to judge whether the host immune system has cleared the infection. The Khan group performed experiments using the pathogen *S.* Typhimurium [43]. After a bacterial challenge, the liver and spleen of mice were aseptically harvested and homogenized. Viable bacterial counts were then collected by plating on LB agar plates. Two different organs were used in order to show comparative results of the bacterial counts. The authors showed that the T3SS and flagella systems are down-regulated when the actin-like protein MreB is not present [43].

In order to determine the bacterial load of *E. coli* and *C. rodentium,* the Cataldi laboratory dissected the gut of EHEC-infected mice [38]. They also performed a bacterial load assay by plating the luminal contents (un-excreted feces) onto growth agar. This study was of interest due to their use of MacConkey agar. This agar is selective for growth of *E. coli* and *C. rodentium* with colonies of those bacteria being visible as pink and other bacteria appearing white. The use of selective agar ensures the bacterial counts are from the infection.

Organ homogenization of lung and spleen samples was used by the Kobets laboratory in a T3SS inhibition assay with *P. aeruginosa* [46]. They found that treatment with a small molecule T3SS inhibitor led to reduced CFU counts. The inhibitor was administered at the time of infection and the following four days. These results showed that colonization was reduced, while inhibitor was given daily. These results contribute to our understanding that T3SS inhibition may be a viable therapeutic strategy.

#### 2.2.2. Whole Animal Homogenization

Whole animal homogenization is typically done with insects and other invertebrates [114,129]. The use of this method allows for calculation of CFUs within the whole animal, leading to a more accurate representation of the total amount of bacteria at time of death. This technique is advantageous because CFU counts may be underestimated when organ homogenization is used. The Attree laboratory used whole animal homogenization in their study of *P. aeruginosa* [129]. They used *Drosophila* and found that a knockout of the T3SS regulator pexsCBA led to less type III secretion. The Hung laboratory performed whole animal homogenization using zebrafish and showed that knockouts of the T3SS structural protein pscD in *P. aeruginosa* led to decreased infection [133].

*G. mellonella* was used by the Venkatesan laboratory to study *Shigella* virulence by comparing wild-type to mutated strains [109]. They tracked the course of infection over time, homogenizing four moth larvae at each timepoint. They pooled together these four live and infected moth larvae and homogenized them in order to obtain bacterial load counts. A library of Shigella mutants was used that had knockouts of individual effector proteins. The authors ultimately showed that multiple effector proteins in *Shigella* are involved in virulence. This example shows the importance of using small animals when doing whole animal homogenization. Large numbers of animals are required to obtain data at all of the desired timepoints, meaning that using larger animals would be impractical.

#### 2.2.3. Sample Collection

Bacterial load methods often require euthanasia or are performed after the death of the animal. This limitation prevents monitoring bacterial loads over time in the same animal. Methods that do not require the death of the animal include collecting samples such as feces [38,134] or vaginal secretions [48]. Samples are usually collected aseptically, meaning they are not collected from the floor of the enclosure, but instead, collected from the animal itself. After collection, the sample is prepared and plated to determine bacterial loads.

The effect of a T3SS inhibitor on acute and chronic *Chlamydia* infection in mice was investigated by the Kobets laboratory [48]. They showed that T3SS inhibition by *N*-(2,4-difluorophenyl)-4-(3-ethoxy-4-hydroxybenzyl)-5-oxo-5,6-dihydro-4*H*-1,3,4-thiadiazine-2-carboxamide, also called CL-55, led to decreased *Chlamydia* shedding in both the upper and lower vaginal canal. When treated daily with CL-55, overall vaginal bacterial cell counts were lowered to that of uninfected mice by day 14. Real time PCR was also performed to show dose-dependence between T3SS inhibitor and chlamydial DNA [48].

### 2.3. Histopathological Observations

Histopathological observations, often called organ scoring, include a wide range of assays that measure specific disease characteristics and symptoms caused by a pathogen. These assays are typically quantified using a numbering system determined by the laboratory that uses them.

#### 2.3.1. *P. aeruginosa*

*P. aeruginosa* infection is characterized by subcutaneous abscess formation [26] or fluid accumulation in the lungs [135] *P. aeruginosa* is often studied in rodent species such as mice and rats, and these symptoms are used to determine the severity of disease. The Berube laboratory used abscess formation in mice as the primary indicator during their screen of potential T3SS inhibitors [26]. Following subcutaneous injection of *P. aeruginosa*, an abscess formed that protruded from the side of the mouse. The length and width of the abcess was measured externally daily in order to track progression of the disease.

Zebrafish can also be used to study *P. aeruginosa*. The Ramakrishnan and Moskowitz groups collaborated to study how the *P. aeruginosa* T3SS interacts with phagocytes during systemic infection [136]. They used transgenic fish that were transparent, along with fluorescently-dyed phagocyes in order to monitor the interaction in real time. During this experiment, they noted that macrophages and neutrophils were the most active and important phagocytes in preventing *P. aeruginosa* infection. They were also able to observe red blood cell aggregation. This is of particular interest, because thrombotic complications are common in humans infected with *P. aeruginosa* [137].

#### 2.3.2. Intestinal Bacteria

Intestinal pathogens such as EPEC, EHEC, and *S.* Typhimurium can be studied by observing damage to, or permeability of, the intestines [36,39,138]. Infections can also be observed by scoring the diarrhea of the animal as the disease progresses. A common scoring method for diarrhea can be seen in Table 2 and is similar to one used by the Waldor group in their studies of EHEC in rabbits [65,66].

Damage to the intestines of mice can be measured by using a permeability assay with the reagent FITC-Dextran [35]. This reagent is a fluorescent compound that only weakly permeates the gut in health mice. When an infection causes damage to the gut, FITC-Dextran can permeate much more readily, allowing the reagent to enter the bloodstream. Blood samples collected from mice can be analyzed for fluorescence and this measurement can be correlated to infection.

The Withey laboratory devised a way to measure diarrhea of zebrafish infected with *Vibrio cholera* [101]. They did so via three methods, the simplest of which was taking the optical density (OD) of the water in which the fish were contained. They also used a variation on the periodic acid Schiff assay in order to determine mucin levels [139]. Mucin secretion is induced by *V. cholera* infection, and therefore is an indicator of infection. Third, they used a standard Bradford assay to estimate overall protein levels in the water [140]. Through these three methods, they were able to quantify the diarrhea of zebrafish and showed dose dependence between diarrhea and challenge by *V. cholera*.

#### 2.3.3. *Bordetella pertussis*

*B. pertussis* infection, also known as whooping cough, is characterized by paroxysmal coughing in humans. Upon infection, this coughing is also present in rats, but not mice [28]. The Horiguchi group monitored the number of coughs of rats over specific time periods after challenge of *Bordatella*. The number of coughs was correlated to the severity of infection [28]. The set-up included a soundproof box that encased a single rat and a recording microphone, as well as food, water, and enrichment. They used this assay to investigate the role of the effector *BspR* (also known as *BtrA*), an effector that negatively regulates the T3SS. Knockouts of this effector led to enhanced infection, but no change in coughing.

### 2.4. Competition Assays

In a competition assay, multiple strains of bacteria are given to an animal in order to determine which is more virulent. These assays are used to obtain a competitive index (CI) in order to compare the virulence of different strains. CIs are calculated as the ratio of mutant to wild-type bacteria in the animal, divided by the ratio of mutant to wild-type bacteria originally used to infect the animal [81,94,141]. A CI greater than 1 means that the mutant strain is more virulent, and a CI less than 1 means it is less virulent than the wild-type.

Chickens were used by the Eade laboratory for competition assay experiments with *Salmonella* strains [95]. *Salmonella* pathogenicity island 1 (SPI-1) is the gene cluster that encodes for the first (T3SS-1) of two T3SSs that are used by pathogenic *Salmonella*. In order to determine the importance of T3SS-1, they created SPI-1 knockout strains. They used both 1:1 and 1:4 ratios of SPI-1 knockout to wild-type pathogen. They found that SPI-1 is required for effective intestinal colonization.

*Xenopsylla cheopis* fleas are a natural host of *Y. pestis* [112,142]. The Fisher laboratory used in vivo and in vitro competition assays to determine the comparative virulence of *Y. pestis* mutants [142]. They knocked out multiple unique virulence factors, most of which are related to biofilm formation. In vitro assays were performed using a 1:1 ratio of wild-type *Y. pestis* to each mutant strain. The bacteria were inoculated into heart infusion broth and their growth was measured over time. It was found that several mutant strains were able to outcompete the wild-type strain for growth, but in vivo testing showed that none of these faster growing strains was more virulent. The combination of in vivo and in vitro assays highlights the importance and necessity of using an animal model to determine virulence.

### 2.5. Virulence Expression/Effector Secretion Assays

The T3SS is responsible for the secretion of effector proteins, and their expression can be correlated to T3SS function and inhibition [11,44,45]. Many T3SS inhibitors have been discovered using in vitro secretion assays [33]. These assays are typically performed by monitoring effector protein release into the supernatant. These assays can also be performed in animals, giving a significantly more realistic model of infection, pharmacokinetics, and immune response. Another strategy for measuring T3SS expression is to monitor the transcription of T3SS genes using reverse transcription PCR [45,143]. This allows for quantification of gene expression for specific effectors or regulatory genes. Bohez and coworkers used real-time PCR to monitor SPI-1 genes in *Salmonella enterica* serovar Enteritidis (*S.* Enteritidis) [143]. They found that a knockout of the T3SS transcriptional regulator hilA in *S.* Enteritidis prevented infection in chickens.

The Giacomodonato laboratory has published multiple papers describing strains of *S.* Typhimurium that produce FLAG-tagged T3SS effector proteins such as SopA, SopB, and AvrA [44,45]. These proteins can be quantified by immunoblotting due to the FLAG tag. They used these strains of *S.* Typhimurium to track the expression of these FLAG-tagged proteins in mice. The authors took samples from the mesenteric lymph nodes that became swollen during infection. This allowed them to observe the functional secretion by the T3SS after taking intestinal fluid samples. The conclusion of these experiments was that SopB and AvrA are secreted by both the T3SS-1 and T3SS-2, meaning that they are important for both initial and systemic *S*. Typhimurium infection.

#### Imaging

Bioluminescent imaging can be used to monitor gene expression of engineered EPEC strains in mice [37]. The Hecht laboratory introduced the *luxCDABE* operon from *Photorhabdus luminescens* into EPEC. This gene cluster produces luciferase, which can be monitored either in vivo or after organs have been harvested. Plasmids containing native effector gene promoters (*LEE4*, *nlaH1*, *nleH2*, and *map*) and *luxCDABE* were created and then introduced into EPEC. *LuxCDABE* was used as a reporter to study the temporal expression for each of the genes: *espF*, *nleH1*, *nleH2*, and *map,* respectively. This allowed the authors to use bioluminescent imaging in mice to approximate the production of each individual effector protein over the course of infection.

A collaboration between the Voelz and Krachler groups used fluorescent imaging of EHEC in zebrafish [29]. They used two different strains of EHEC that made the fluorescent protein mCherry. In the first, the production of mCherry was constitutive, and in the second, it was used as a reporter for the expression of the LEE1-encoded regulator Ler. This system allowed them to image and monitor localization, colonization levels, and expression of the T3SS. They used this system to show the importance of gut commensal bacteria in preventing EHEC infection.

### 2.6. Vaccines and Immunizations

Vaccination studies are commonly done in mice [40,42,49], rabbits [65,66], cattle [81], and primates [86,91,93]. These studies can demonstrate the power of inhibiting the T3SS by using antibodies that recognize T3SS effectors or structural components. One of the most notable instances of recent vaccine research occurred in the Pasetti laboratory [49]. They have developed a combined YopB and LcrV subunit vaccine that acts against *Yersinia enterocolitica*, a pathogen that causes severe enteric infection in young children and infants. The vaccine is also effective against *Y. pestis.* Both infant and adult mouse models were used, with >90% of adults and 60% of infants protected against infection by *Y. enterocolitica*.

Vaccine studies have been performed with mice to study the prevention of vaginal infection with *Chlamydia muridarum* [144]. Kobets and coworkers have developed a vaccine containing the T3SS needle tip protein, CdsF, in order to provide protection. They used an adenoviral vector in order to administer the CdsF antigen. Anti-CdsF antibodies of the IgG2a and IgG1 isotypes were detected and quantified via enzyme-linked immunosorbent assay (ELISA). They also intravaginally challenged the mice with *C. muridarum* and tested for bacterial loads via vaginal swabs on days 3, 6, 9, 15, and 20 post-infection. During the infection challenge, they also tracked the number of CD4^+^ and CD8^+^ T cells, common markers of an immune system actively clearing infection. These assays showed that CdsF-based vaccines have potential to prevent infection by *C. muridarum*.

Non-human primates are the most accurate model of the human immune system for vaccines. The Venkatesan laboratory studied live *Shigella sonnei* vaccine strains in rhesus macaques [91]. They modified the genome of *S. sonnei* in order to make a non-virulent strain of the pathogen to use as a live vaccine. Previously, live attenuated vaccines lacking only one virulence gene, *Vir*G, have been tested in human clinical trials [145]. These were unable to progress past phase 1 due to side effects, as 20% of the volunteers developed fever. The Venkatesan laboratory has created a strain of *S. sonnei* which lacks *Vir*G, a gene for an autotransporter, as well as two other virulence genes, *sen*A and *sen*B, which encode effectors that are secreted by the T3SS [91]. This vaccine strain showed an increase in blood leukocyte counts, which shows that an immune response was stimulated, with minimal side effects in the macaques.

#### Maternal Vaccination

Maternal vaccination is a variation of the typical vaccine or immunization assay. The Merkel laboratory used baboon females that were pregnant and immunized them with a vaccine for *B. pertussis* [86]. They then waited until the infant baboons were born and performed antibody testing on the infants to determine how effective the vaccine was at transmitting from the mother to child, either in the womb pre-birth or via nursing post-birth. They also gave the infants a *B. pertussis* challenge. Children from unvaccinated mothers had more severe symptoms after infection than those whose mothers were vaccinated, showing that maternal vaccination was effective.

The Gerdts and Elahi groups investigated maternal vaccination of sows and the effect on their piglets with respect to *B. pertussis* [79]. They used heat-inactivated bacteria to vaccinate pregnant sows, nursing sows, and piglets still suckling. They showed that direct vaccination of piglets was not enough to induce high-level production of antibodies and did not protect against *B. pertussis* challenge. Maternal vaccination, either before or after birth, was shown to provide some level of protection, as monitored by antibody levels. They also showed the importance of breast feeding by comparing the antibody levels of piglets who were born to vaccinated mothers, but breast fed by non-vaccinated mothers to the levels of piglets who were born to non-vaccinated mothers, but breast fed by vaccinated mothers. They showed that vaccination of mothers during nursing is more important and provides more protection to the piglet regardless of vaccination before birth.

### 2.7. Surgical Interventions

Surgical techniques, such as organ harvesting, often require the euthanasia of the animal prior to assay completion. Surgical interventions differ from these techniques in that the animal is kept alive for longer time periods to observe infection. Surgical interventions, such as the traditional ligated ileal loop assay, often keep the animal anesthetized for the duration of the experiment and require euthanasia afterwards due to the damage done during the intervention. Other examples, such as the xenograft, allow the animal to recover and assays to be performed after the intervention. The ligated intestinal loop and xenograft assays will be discussed in this section.

#### 2.7.1. Ligated Intestinal Loops

Ligated ileal loops are the most common surgical intervention in the field of bacterial pathogenesis. They are routinely used with cattle [82,83,146,147,148] and rabbits [31,149], but have also been used in pigs [75] and rodents [23,150] In a ligated ileal loop assay, the small intestines, typically the ilium, of an animal are surgically sealed off into sections called loops. The large intestine, or colon, can also be used to create ligated loops. The number of loops varies based upon the size of the animal; rats typically have 2–3 sections throughout the entire intestine while, adult cattle may have as many as six within the ilium itself. Each section is then treated with the pathogen and/or the compound of interest. In traditional ligated loop assays, the animal is monitored for up to 24 h, at which time they are sacrificed, and results recorded. This style of assay is also performed ex vivo, or when the animal is euthanized near the time of infection. Then, the loops are removed and placed into an incubator for the remainder of the experiment. This method allows for researchers to test multiple bacterial strains or compounds within the same animal. This decreases the error associated with using multiple animals, such as differences in microbiota.

The use of ligated intestinal loops was originally used in rabbits starting in the 1950s [151]. Since then, many improved versions of the assay have emerged. The Tang and Sansonetti groups used a traditional rabbit ileal loop in order to demonstrate the importance and function of *spa32* and *spa33*, virulence genes which initiate T3SS secretion in the presence of certain oxygen concentrations, in *Shigella* infections [27]. They used the ileal loops to determine a competitive index comparing different knockout strains and mutants. The ileal loops were formed, inoculated, and placed back into the abdominal cavity. After 18 h, the rabbits were sacrificed and the loops were weighed to determine fluid accumulation, the contents plated for bacterial counts, and then stained for histopathological observations. The conclusion of the paper was that in the anaerobic lumen of the intestine, the T3SS will not be fully expressed, but the mucosal surface, which is more aerobic in comparison, allows for complete activation of the T3SS.

The Finlay group used a bovine ileal loop model to study *S.* Typhimurium [83]. They developed an ileal loop model based upon a sheep model using intestinal clamps and removable silk sutures to separate the sections of intestine. These clamps and sutures could be removed, meaning that the animals did not have to be sacrificed to obtain data. The animals can also be studied after the sutures and clamps have been removed, allowing for study of histopathological observations of later stages of infection. In a traditional ileal loop assay, euthanasia takes place within 24 h post-infection. With the reversable loop assay, the authors were able to observe histopathological effects up to 5 days post-infection. The authors validated this novel assay using T3SS-1 and T3SS-2 knockouts and comparing the results to the well-described murine model. The Stevens laboratory also performed this removable ligated ileal loop assay in cattle with the pathogen *Salmonella* enterica serovar Dublin (*S.* Dublin), a strain that can infect both humans and cattle. They showed that T3SS-1 of *Salmonella* is required for lymphatic translocation, but T3SS-2 is not [82].

Guinea pig ileal loop assays were used by the Shao and Lu groups to study shigellosis [150]. Because guinea pigs are so small, the colon, as well as the ilium, was ligated. They formed two ileal loops and two distal colon loops, each only 2–3 cm in length. They used these loops to investigate the role of human [alpha]-defensin 5 (HD5), an antimicrobial peptide. They showed that HD5 enhances *Shigella*’s ability to adhere and colonize the gut. This shows that *Shigella* can become more infective in the presence of a host immune response.

#### 2.7.2. Xenotransplant Models

Xenotransplant is a surgical intervention where an organ from a species different than the recipient is used as a replacement. In infectious disease research, the donor organ is often human or primate. During their studies of T3SS-dependent attaching and effacing lesions, the Shpigel laboratory replaced a section of the mouse intestine with a section of pediatric human gut [24]. They did so in order to test EPEC, which normally does not infect mice, but is capable of infecting humans. The authors were able to reliably cause infection using this xenotransplant model. Having human gut within mice allows EPEC to be tested and function as a realistic model of human infection.

## 3. Conclusions

Most T3SS inhibitors to date have been identified and studied using in vitro assays. Notwithstanding these contributions, in vivo models are needed in order to prove the effectiveness of T3SS inhibitors. Due to the importance and potential of T3SS inhibition as a therapeutic strategy, researchers should be aware of in vivo assays available in the field.

## Figures and Tables

**Table 1:** Selected references for common type III secretion system (T3SS)-utilizing bacteria and their animal models.

	*E. coli*	*Salmonella* spp.	*Pseudomonas* spp.	*Shigella* spp.	*Bordetella* spp.	*Vibrio* spp.	*Chlamydia* spp.	*Yersinia* spp.
Mouse	[35,36,37,38]	[39,40,41,42,43,44,45]	[46]	[47]	[11]	[12]	[48]	[49,50]
Rat	[23]	[51]	[52]	[53]	[28]	[54,55]	[56]	[57]
Guinea Pig	[58]	[59]	[60]	[61]	[62]		[63]	[64]
Rabbit	[65,66]	[59]	[67]	[27]	[68]	[69]	[70]	
Cat and Dog	[71,72]		[73]		[74]			
Pig	[75]	[76]	[77]	[78]	[79]		[80]	
Cattle	[81]	[82,83]					[84]	
Baboon	[85]				[86]		[87]	
Macaques	[88]	[89]	[90]	[91]			[92]	[93]
Chicken	[94]	[95]		[96]			[97]	
Zebrafish	[29]	[98]	[99]	[100]		[101]		
Worms	[102]	[103]	[104]	[105]		[106]		[103]
Wax Moth	[107]	[59]	[108]	[109]		[110]		[111]
Flea								[112]
Fly	[113]	[114]	[113]			[115]		
Amoeba		[116]	[117]	[118]		[31]		

**Table 2:** Diarrhea scoring in rabbits.

Score	Symptoms	Action to Take
1—None	No diarrhea (normal pellets are dark green, hard, and formed)	None required
2—Mild	Mix of soft yellow–green unformed and formed pellets, resulting in light staining of the hind legs	Increase monitoring of animal
3—Severe	Unformed or liquid stool, resulting in significant staining of the perineum and hind legs	Animal should be euthanized
